# Carbon dot/cellulose nanofiber composite: Dataset for its water treatment performance

**DOI:** 10.1016/j.dib.2021.106760

**Published:** 2021-01-14

**Authors:** Jungbin Ahn, Sewon Pak, Hyungsup Kim

**Affiliations:** Department of Organic and Nano System Engineering, Konkuk University, Seoul 05029, Republic of Korea

**Keywords:** Carbon dot, Cellulose nanofiber, *In-situ* synthesis, Morphological durability, Water treatment membrane

## Abstract

Carbon dot (CD) obtained via one-step hydrothermal carbonization was attached to cellulose nanofiber (CNF) via physical blending and *in-situ* synthesis. The data represent the morphological, chemical and optical differences of the samples, according to the amount and introducing method of CD. The morphological durability of the samples was also shown. The water treatment membrane performance was analysed using methylene blue as a representative pollutant. The related article was published in Carbohydr. Polym. 255 (2021) 117387.

## Specifications Table

**Table:** 

Subject	Physics, Chemistry, Material science
Specific subject area	Cellulose nanofiber, Carbon dot, Membrane, Composite
Type of data	Table, figure, graph, image
How data were acquired	AFM (XE-70, Park Systems, Korea), XPS (K-alpha, Thermo Scientific), UV spectrometer (Cary 60 UV/vis spectrophotometer, Agilent Technologies), TGA (TGA 8000, Perkin Elmer, USA) Digital camera (G10, Canon), SEM (SU8010, Hitachi, Japan)
Data format	Raw, analyzed
Parameters for data collection	The CD embedded films were prepared via simple dead-end filtration method and dried in vacuum oven before characterized. All the characterizes were conducted without any treatment.
Description of data collection	Samples for AFM were prepared by simple drop casting of 0.001 wt% suspensions, which was dried overnight in ambient conditions. FT-IR spectra was obtained with 32 scans, 4 cm^−1^ of resolution. UV absorption of the prepared suspensions were obtained using 0.5 wt% suspension. TGA data were collected with 5 mg of film loaded, and the temperature were elevated by 10 °C/min for all the samples. Surface SEM images of the samples were obtained by applying 3 kV voltage.Membrane test results were obtained using Amicon 8050, Milipore, USA.
Data source location	Konkuk University, Seoul, Korea
Data accessibility	All raw data are available in this article and in https://data.mendeley.com/datasets/43frjz5y86/2
Related research article	These data are supplementary to the article: Jungbin Ahn, Sewon Pak, Younghan Song, and Hyungsup Kim, *in-situ* synthesis of carbon dot at cellulose nanofiber for durable water treatment membrane with high selectivity, Carbohydr. Polym. 255 (2021) 117387.
	https://doi.org/10.1016/j.carbpol.2020.117387

## Value of the Data

• This data can help for the understanding of difference of the composite between the CD-CNF blended and *in-situ* synthesized CD@CNF composite and their resulted membrane performance.
• The *in-situ* synthesis of CD on the surface of CNF increased the interaction between the CNFs and enhanced the morphological durability, as well the pure water flux.
• The data exhibit the possibility of the prepared CD@CNF composite for highly effective water treatment membrane.

## 1 Data Description

**Fig. 1:**
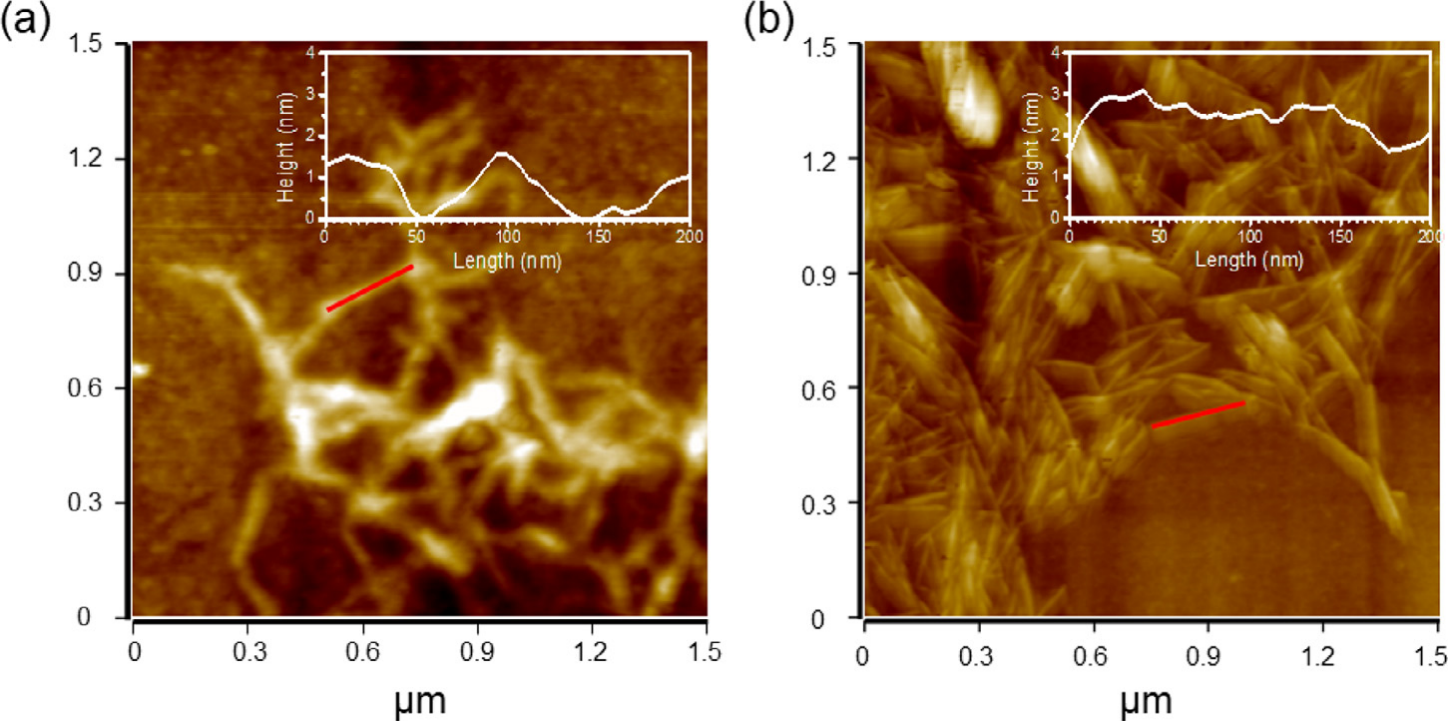
AFM images of (a) CNF and (b) CD@CNF.

Fig. 1 shows the AFM images of the samples. The CD@CNF showed increased height compared to CNF (Fig. 1 (a)).

**Fig. 2:**
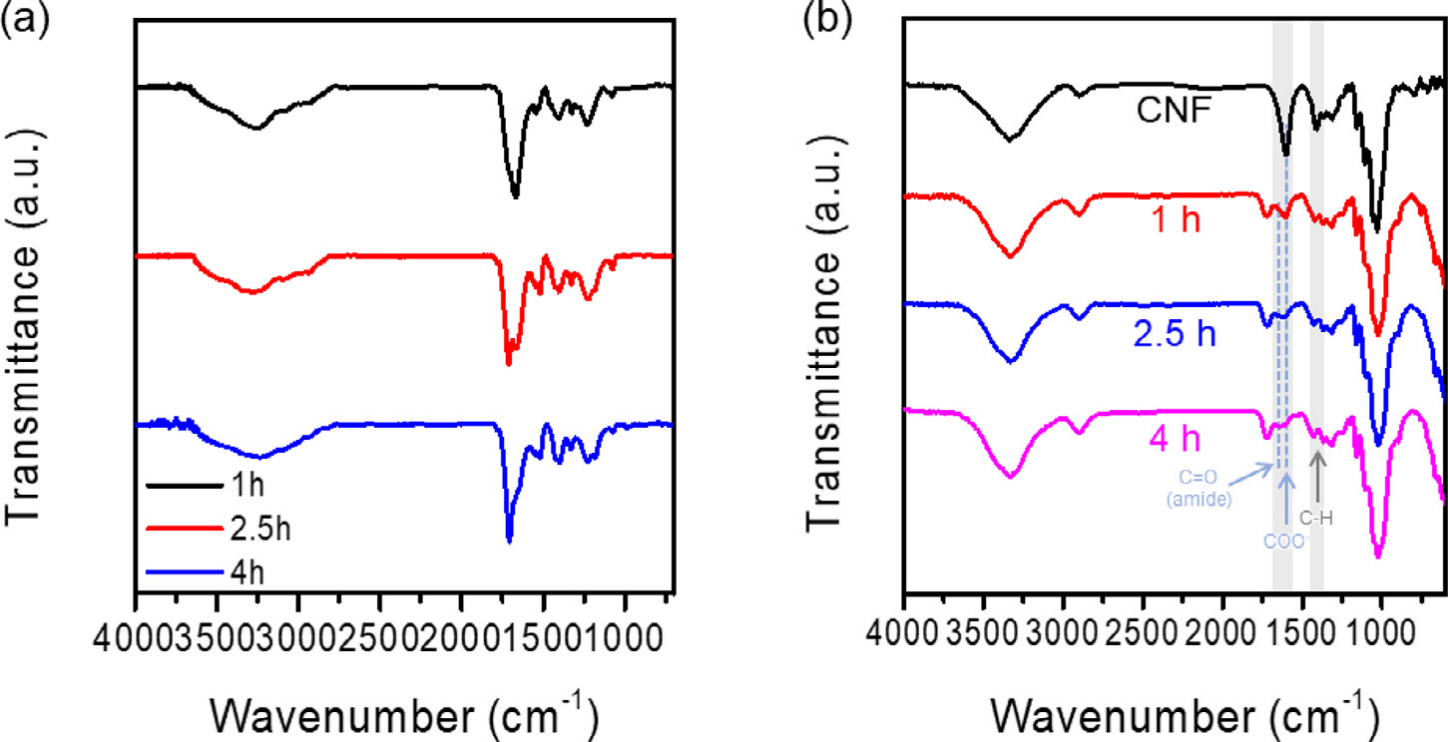
FT-IR spectra changes of (a) CD and (b) CD@CNF according to the HTC time.

In CD@CNF samples, the peak for C=O was increased and the peak for COO^−^ was decreased according to the increase of HTC time. (spectra of 4 h CD and 4 h CD@CNF were reproduced from Carbohydr. Polym. 255 (2021) 117387).

**Fig. 3:**
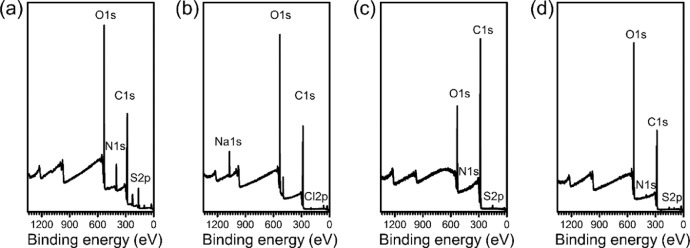
XPS survey spectra of (a) the CD, (b) the CNF, (c) CD-CNF blended and (d) CD@CNF film.

Fig. 3 described the XPS survey spectra of the samples.

**Fig. 4:**
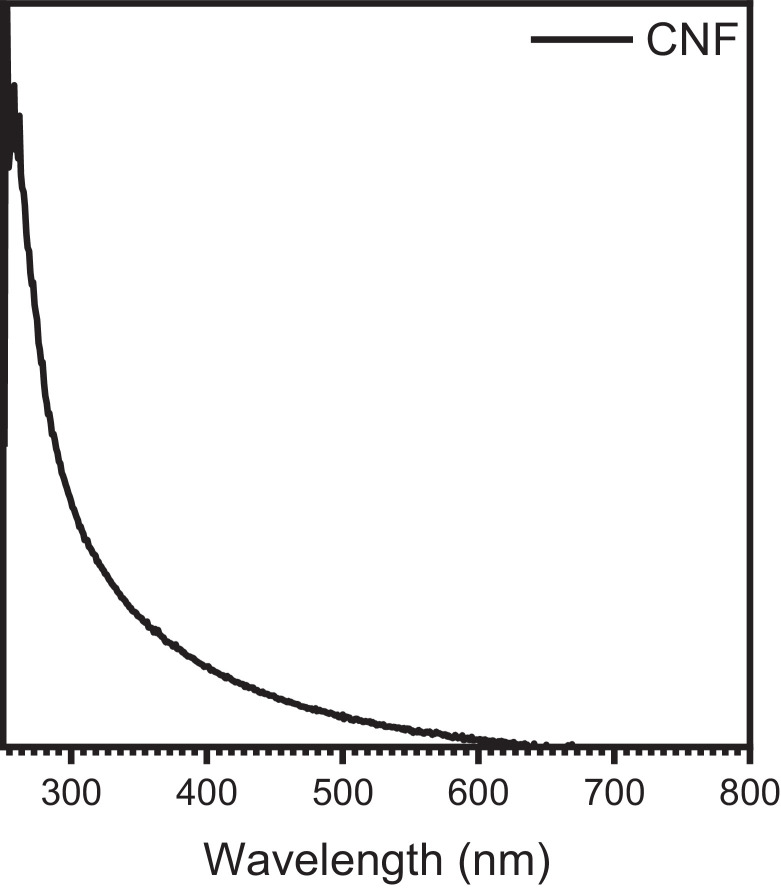
UV absorption curve of the CNF suspension.

Fig. 4 exhibited the UV absorption curve of the CNF suspension.

**Fig. 5:**
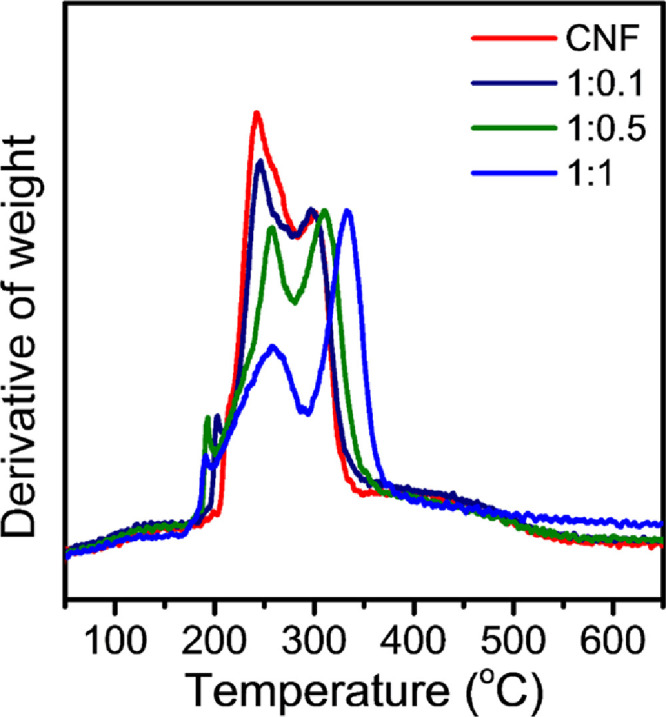
DTG curves of the CD-CNF blended films with different CD contents (1:0, 1:0.1, 1:0.5 and 1:1, with respect to the CNF weight) (CNF curve was reproduced from Carbohydr.Polym. 255 (2021) 117,387).

Different contents of the CD were blended with the CNF suspension to study the effect of CD on the thermal behavior of the films. The DTG peak intensities of the samples were normalized by the degradation peak intensities corresponding to cellulose. The degradations of the CDs were observed around 190 °C, independently of the CD contents. However, the degradations of the CNF were considerably changed, as the CD content was increased. With higher contents of the CD, the degradation temperatures were shifted toward higher temperatures. The increase was owing to the presence of aromatic structures in the CDs, acting as a thermal barrier. It made the CNF more stable in elevated temperature. Another change was observed according to the CD contents. The peak intensities corresponding to glucuronate group degradation were gradually decreased with the increased CD contents. As the CD had secondary interactions mainly with the carboxylate groups of the CNF, it increased the thermal resistance Fig 5.

**Fig. 6:**
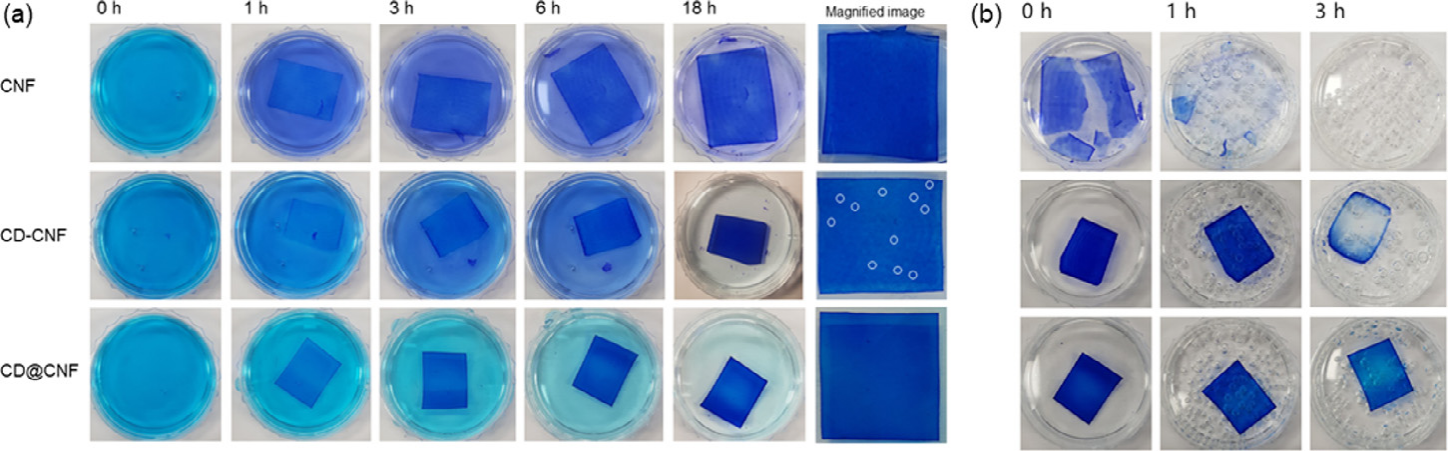
Photographs of the films along the time (a) immersed in MB solutions and the magnified images of the films after 18 h, (b) immersed in hydrogen peroxide solutions with 365 nm of UV light irradiation.

Fig. 6(a) shows the morphological changes of the films in MB solutions. The films in MB solution were stored in darkroom without stirring. The CNF was extensively swollen while the others were not. All the film showed high absorbing capacity of MB. As shown in the figure, the colored spots were unevenly observed in the CD-CNF film (white color). It indicates inhomogeneous state of the film which might be resulted from the CD aggregation. Meanwhile, the CNF and the CD@CNF films were evenly dyed by MB without any spots. After the full swelling for 18 h, the films were immersed in hydrogen peroxide solution (30 w/w% in H_2_O) with the 365 nm of UV light (4 W) irradiation (Fig. 6(b)). The CNF film lost its morphological structure in a short time. Compared to that, the CD@CNF film successfully maintained its dimension up to 3 h.

**Fig. 7:**
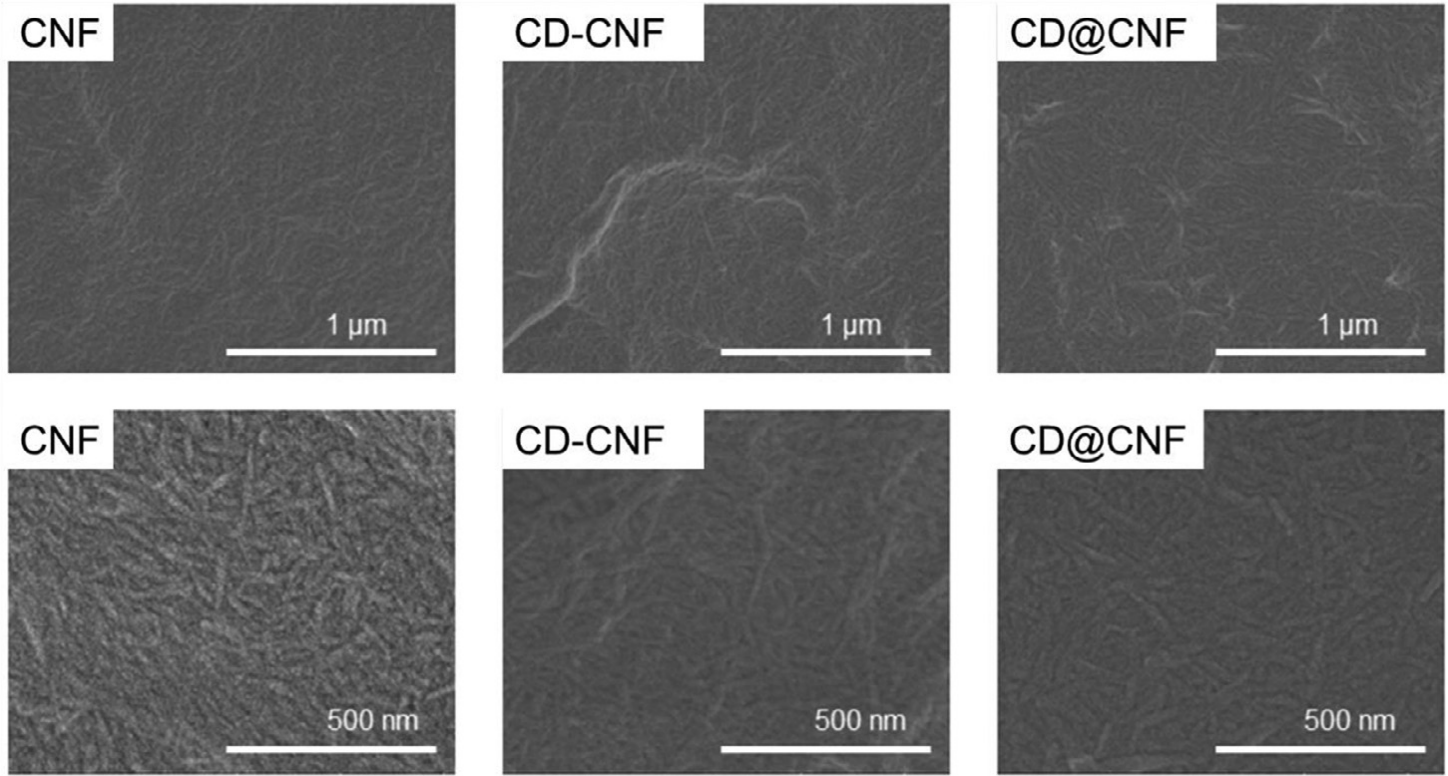
SEM images of the CNF, CD-CNF and CD@CNF membrane (20 gsm) and their high magnification images.

Fig. 7 shows the SEM images of the samples.

**Fig. 8:**
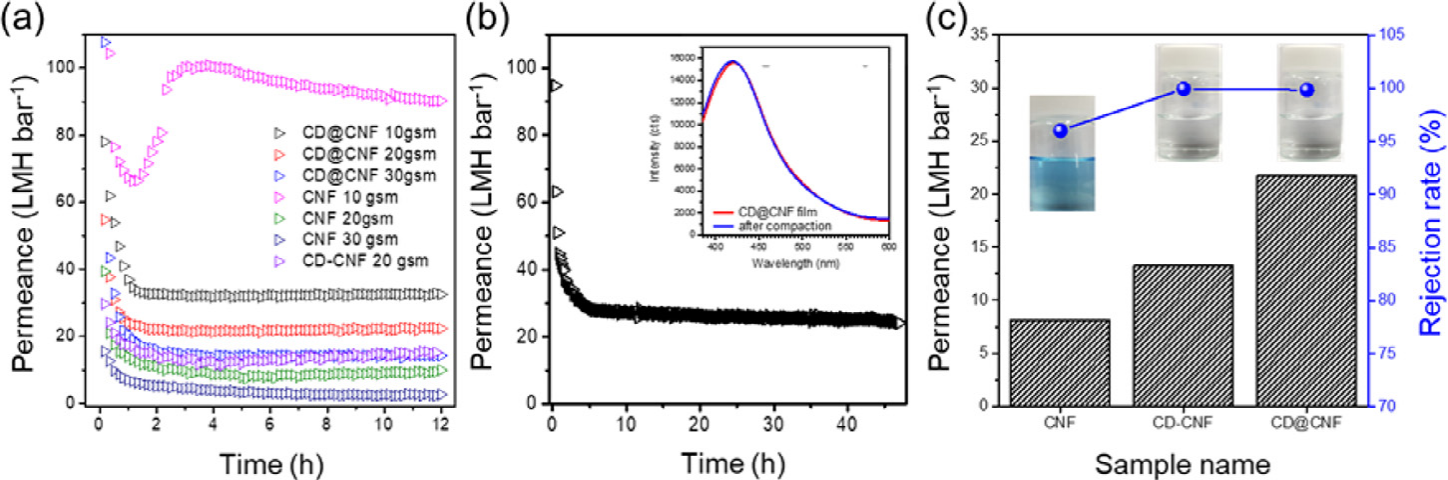
(a) Pure water flux curves versus time of samples. (b) Pure water flux of 20 gsm loaded CD@CNF for 48 h (inserted graph displays the PL intensity of CD@CNF before and after water pressure loaded) (c) Pure water flux of 20 gsm loaded samples and their MB rejection.

Fig. 8(a) shows the pure water flux curves of the samples along the time. The 10 gsm loaded CNF showed unstable water permeability due to inferior mechanical properties. As like, the 20 gsm loaded CNF also exhibited slight increasement of permeability along time. In case of the 30 gsm loaded CNF, the membrane showed slow and continuous decrease of water permeability, indicating the membrane was steadily compacted in the observation time. Although the CD introduction enhanced the water permeability of the membranes, the CD-CNF blended membrane still exhibited unstable water permeability along the time. The *in-situ* synthesized CD@CNF membrane showed stable permeability without flux drop after 2 h, with regardless of loading amount. The pores in the membrane were maintained without significant structural collapse or deformation (Fig. 8(b)). The inset of the Fig. 8(b) shows the PL intensity of the CD@CNF film with and without 48 h of water pressure (1 bar). These show the CD was well-embedded in the CD@CNF film without any leach out or separation from CNF. In Fig. 8(c), the CD-CNF and the CD@CNF membranes showed high rejection rate while that of the CNF were relatively low. The 20 gsm CNF membrane was not mechanically strong enough to maintain the structure under 1 bar of pressure and resulted the low rejection rate. In spite of that, the CD-CNF membrane showed increased rejection rate which proves the strong affinity between the CD and MB. Among the prepared samples, the CD@CNF membrane showed the highest water flux and rejection rate due to the CD-enveloped CNF structure.

**Fig. 9:**
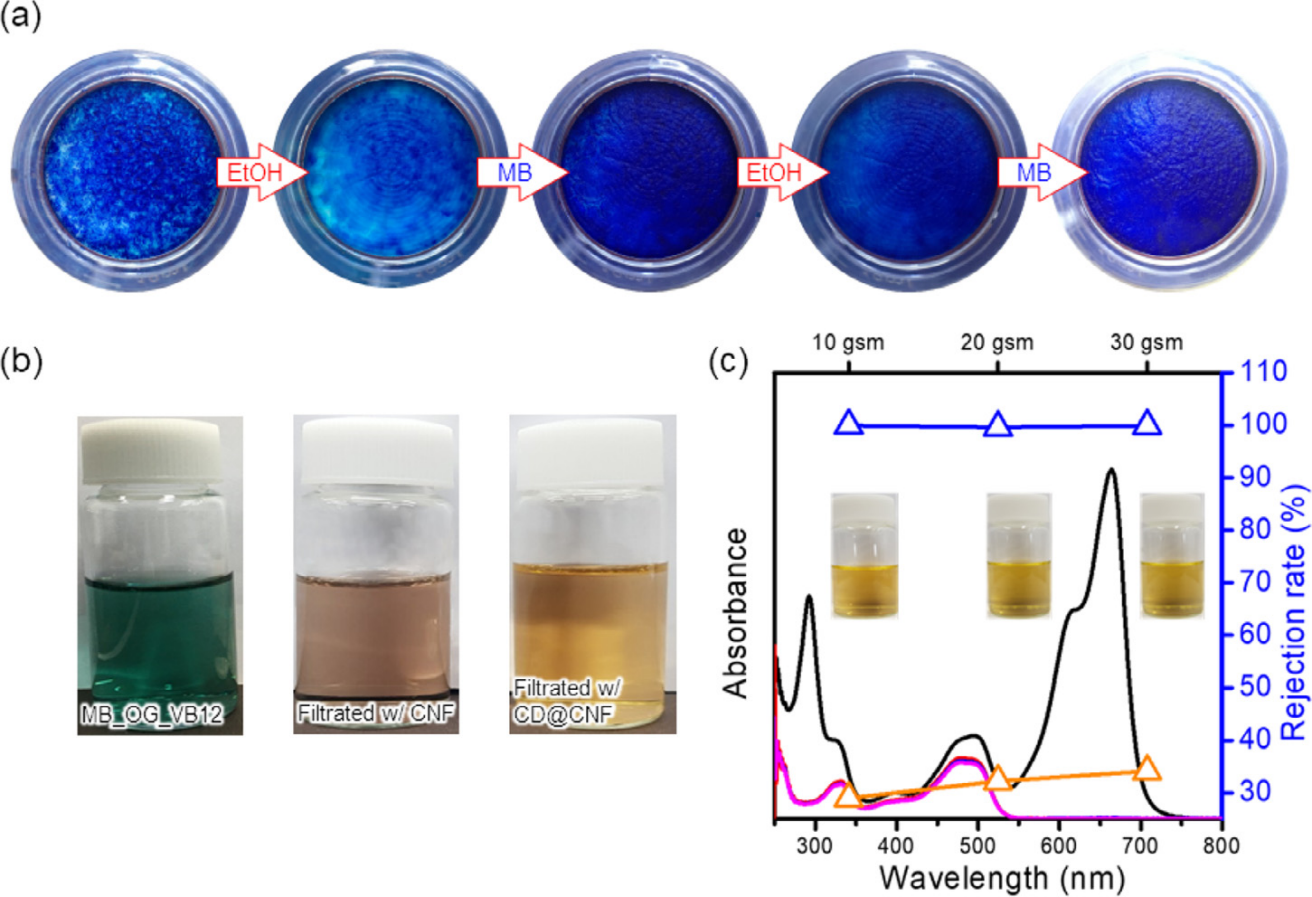
(a) Digital images of the CD@CNF membrane for the recycling test. The membrane was washed with 50 ml of ethanol before the next cycle. (b) The digital images of the mixed dyes solution before and after rejected with CNF and CD@CNF and (c) Selective dye removal performance of CD@CNF membrane according to the loading amounts.

The CD@CNF membrane showed recycle-ability toward MB rejection up to third cycle, having 95% of rejection rate (Fig. 9(a)). Moreover, the CD@CNF membrane showed better cationic dye selectivity than the CNF membrane even in the presence of anionic and neutral dyes (Fig. 9(b)). The CD@CNF membrane showed good dye selectivity regardless of loading amounts (Fig. 9(c)). The CD@CNF membrane selectively rejected cationic dye from the mixed solution of MB and OG (Table 1).

**Table 1:** Dye rejection performance of CNF membrane (20 gsm) on differently charged dyes (10 ppm).

Dyes/CD@CNF	Charge	Molecular size (nm)	Rejection (%)
Vitamin B12	Neutral	1.874	18.89
Orange G	Negative (−)	0.905	4.15
Methylene Blue	Positive (+)	1.359	96
VB12+OG+MB	VB12 (20.1%), OG (23.2%), MB (99.1%)		

## 2 Experimental Design, Materials and Methods

### 2.1 Synthesis of carbon dots (CD) and CD@CNF composite

0.5 mmol of glutathione (GT, Sigma Aldrich Co.) and citric acid (CA, Sigma Aldrich Co.) were dissolved in 30 ml distilled water for the CD synthesis. The solution was kept at 120 °C for 4 h. The obtained mixture was dialyzed against water (0.5–1 kDa, Biotech CD tubing) for 24 h. For the CD@CNF composite synthesis, the GT and CA were carefully dissolved in 35.5 g pf CNF suspension (1.8 wt% in water, carboxylate content: 1.7 mmol/g). The mixture was hydrothermally treated under the same condition with the CD. For the comparison, the CD was physically blended with CNF, by the ratio of 1:1 based in the weight percent and named as CD-CNF.

### 2.2 Characterization

To confirm the envelopment of the CD on the CNF surface, AFM images of the CNF and the CD@CNF were obtained using 0.01 wt% suspensions. X-ray photoelectron spectroscopy (XPS, K-alpha, Thermo Scientific) were carried out to characterize the chemical structure of the samples. UV–vis absorption spectroscopy (Cary 60 UV/vis spectrophotometer, Agilent Technologies) were used for optical property analysis and the thermal degradation behaviors of the samples were analyzed using a thermogravimetric analyzer (TGA 8000, PerkinElmer, USA). SEM images were obtained using SU8010, Hitachi, Japan. The membrane performance of the samples were evaluated by filtering distilled water, or organic dye solutions (Methylene blue (MB, 82% dye contents), Orange G (OG, 80% dye contents) and Vitamin B12 (VB12, 98% dye content)) with dead-end filtration (Amicon 8050, Milipore, USA), by loading on filter paper (filter paper No. 6, Advantec, Japan).

## Ethics Statement

This work did not involve human subjects or animal experiments.

## CRediT author statement

**Jungbin Ahn:** Conceptualization, Methodology, Formal analysis, Visualization, Writing – original draft, Funding acquisition, Project administration; **Sewon Pak:** Validation, Formal analysis; **Hyungsup Kim:** Conceptualization, Supervision, Writing – review & editing, Funding acquisition, Project administration.

## Declaration of Competing Interest

The authors declare that they have no known competing financial interests or personal relationships which have or could be perceived to have influenced the work reported in this article.

